# Supplementary material for: Meta-analysis of multidecadal biodiversity trends in Europe
Source: Nat Commun. 2020 Jul 13;11:3486. doi: 10.1038/s41467-020-17171-y (PMC7359034; doi:10.1038/s41467-020-17171-y)
Supplement: Supplementary file 1 — Supplementary Information [file 41467_2020_17171_MOESM1_ESM.pdf]

Meta-analysis of multidecadal biodiversity trends in Europe

Pilotto *et al*. Nature Communications

SUPPLEMENTARY INFORMATION

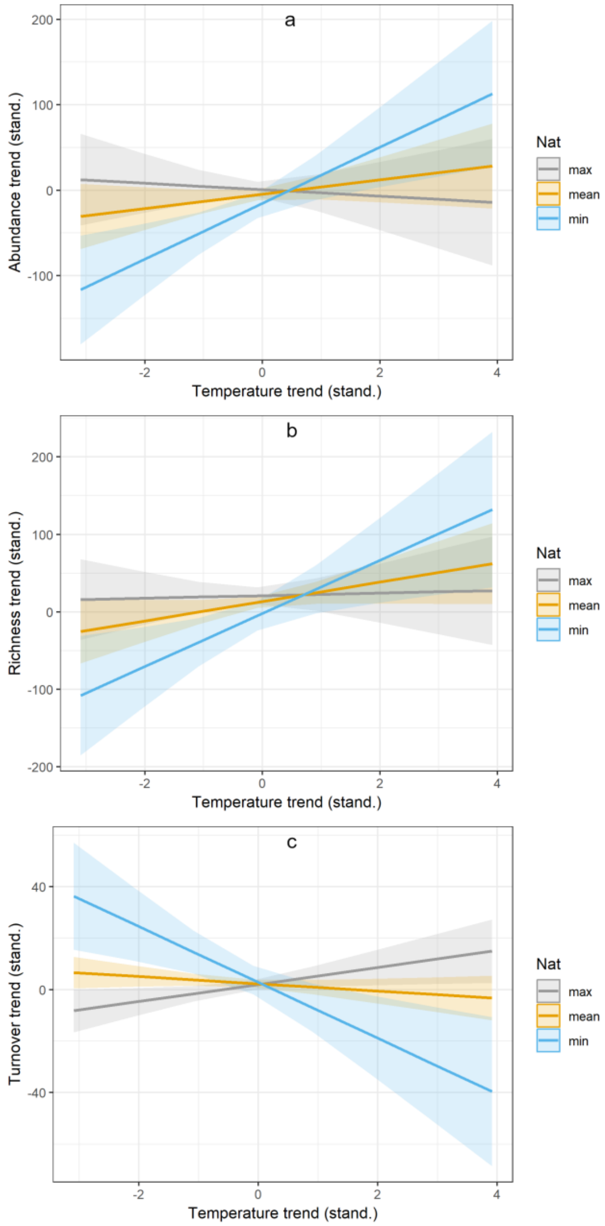

Supplementary Figure 1. Effect of site naturalness and temperature trends on biodiversity trends. Interactions between site naturalness and temperature trends in driving the trends in abundance (a), richness (b) and turnover (c), as resulting from meta-analysis mixed models, based on S-statistics (for further details see methods). Source data are provided as a Source Data file.

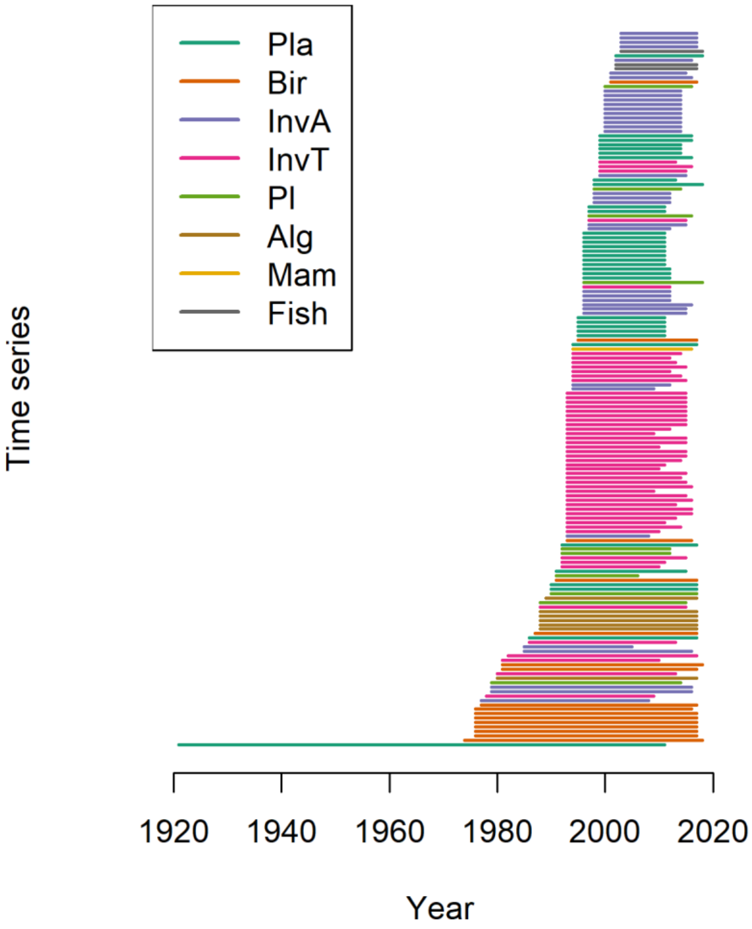

Supplementary Figure 2. Temporal scope of the study. Overview of the temporal distribution and span of the studied time series. Pla: terrestrial plants; Bir: birds; InvA: aquatic invertebrates; InvT: terrestrial invertebrates; Pl: plankton; Alg: benthic algae; Mam: mammals; Fish: fish. Source data are provided as a Source Data file.

Supplementary Table 1. Studied time series. Distribution of time series among biogeoregions, realms and taxonomic groups. MA: marine and transitional zone; FW: freshwater; TE: terrestrial.

	Adriatic			Alpine			Atlantic			Black Sea			Boreal			Continental			Mediterranean			North Sea			Pannonian		
	MA	FW	TE	MA	FW	TE	MA	FW	TE	MA	FW	TE	MA	FW	TE	MA	FW	TE	MA	FW	TE	MA	FW	TE	MA	FW	TE
Benthic algae	-	-	-	-	6	-	-	-	-	-	-	-	-	1	-	-	-	-	-	-	-	-	-	-	-	-	-
Birds	-	-	-	-	-	-	1	-	3	4	1	-	-	-	-	-	4	-	1	-	1	1	-	-	-	-	-
Fish	-	-	-	-	-	-	-	2	-	-	-	-	-	-	-	-	-	-	1	-	-	-	-	-	-	-	-
Aquatic invertebrates	-	-	-	-	3	-	1	8	-	-	-	-	-	14	-	-	4	-	1	-	-	6	-	-	-	-	-
Terrestrial invertebrates	-	-	-	-	-	3	-	-	34	-	-	-	-	-	14	-	-	2	-	-	-	-	-	-	-	-	-
Mammals	-	-	-	-	-	-	-	-	1	-	-	-	-	-	-	-	-	-	-	-	-	-	-	-	-	-	-
Plankton	1	-	-	-	4	-	-	1	-	-	-	-	-	-	-	-	2	-	1	1	-	-	-	-	-	-	-
Plants	-	-	-	-	-	17	-	-	5	-	-	-	-	-	3	-	-	5	-	-	3	-	-	-	-	-	1

Supplementary Table 2. Influence of climatic trends and site characteristics on biodiversity trends. Results of the information-theoretic approach for meta-analysis mixed model selection and multi-model inference, showing the model-averaged coefficients (estimate and 95% C.I.) and the relative importance (sum of Akaike weights, see main text for explanation) of each explanatory variable.

**Abundance (average variation explained: 11%):**				
	**Estimate**	**C.I. 95%**		**Importance**
Intercept	-3.39	-9.33	2.55	1.00
Temperature	7.32	-0.30	14.94	1.00
Naturalness	7.97	1.82	14.11	1.00
Latitude	-0.49	-2.67	1.70	0.21
Study length	-0.35	-2.58	1.87	0.17
Precipitation	0.28	-1.59	2.15	0.17
**Richness (average variation explained: 27%):**				
	**Estimate**	**C.I. 95%**		**Importance**
Intercept	14.13	7.10	21.16	1.00
Temperature	14.44	6.06	22.83	1.00
Longitude	12.75	4.84	20.66	1.00
Naturalness	1.77	-4.12	7.66	0.33
Latitude	0.82	-2.43	4.07	0.21
Elevation	-0.70	-3.66	2.25	0.20
Precipitation	0.55	-1.85	2.96	0.12
Study length	0.36	-1.48	2.21	0.10
**Diversity (average variation explained: 10%):**				
	**Estimate**	**C.I. 95%**		**Importance**
Intercept	6.28	0.20	12.36	1.00
Longitude	11.24	4.54	17.94	1.00
Precipitation	-6.84	-16.14	2.46	0.87
Elevation	-3.76	-10.31	2.80	0.73
Study length	1.97	-4.03	7.96	0.36
Latitude	-0.24	-1.44	0.96	0.10
Temperature	0.15	-0.79	1.10	0.09
**Turnover (average variation explained: 67%):**				
	**Estimate**	**C.I. 95%**		**Importance**
Intercept	2.02	0.63	3.40	1.00
Elevation	1.22	0.23	2.20	1.00
Longitude	-1.04	-3.31	1.23	0.59
Latitude	0.55	-0.81	1.90	0.52
Precipitation	0.44	-0.78	1.66	0.46
Naturalness	-0.50	-2.15	1.14	0.33
Temperature	0.20	-0.51	0.91	0.29
Study length	0.02	-0.10	0.14	0.07

Supplementary notes:

We ran a sensitivity analysis to assess the effect of the unbalanced design on the results of the meta-analysis mixed models. We randomly sampled the over-represented biotic groups within each biogeoregion to the maximum number of time series available for the other biotic groups within that biogeoregion (see Supplementary Table 1). For example, we randomly sampled eight Atlantic terrestrial invertebrate time series (out of 38), as aquatic invertebrates (the second most represented biotic group for the Atlantic region) had eight time series (Supplementary Table 1). We repeated the random sampling 5 times and, with those data, we re-run the meta-analysis mixed models for biogeoregions, as described in the main text. The same procedure was applied for the over-represented biogeoregions within each biotic group. The list of randomly sampled biotic groups and biogeoregions is reported in Supplementary Table 3.

Eventually, 96 out of 100 runs showed the same results as the ones obtained for the full analysis that is presented in the main text (Supplementary Table 3). The only four exceptions were found for: terrestrial invertebrate richness (run 5), terrestrial invertebrate turnover (run 1) and abundance in the Atlantic region (runs 3 and 5; Supplementary Table 3). Due to such a high level of concordance, we are confident that our results are not driven by the most-represented biotic groups and biogeoregion, and are thus robust against unbalanced design.

Supplementary Table 3. Sensitivity analysis. The table reports the results of meta-analysis mixed models for biotic groups and biogeoregions, after five random sampling (runs 1 to 5) of the most-represented biotic groups and biogeoregions. Blue: significantly increasing trends (*p* ≤ 0.05); orange: significantly declining trends (*p* ≤ 0.05); black: no significant trends (*p* > 0.05); italics and red: results that contrast those of the full analysis as reported in the main text.

			**Run 1**		**Run 2**		**Run 3**		**Run 4**		**Run 5**	
**Random sampling:**	**Model results for BIOTIC GROUP:**	**Metric:**	**z**	**p**	**z**	**p**	**z**	**p**	**z**	**p**	**z**	**p**
Boreal aquatic invertebrates (n = 8, out of 14)	Aquatic invertebrates	Abundance	0.15	0.880	0.12	0.910	0.08	0.940	0.01	0.990	0.02	0.980
		Richness	2.00	0.044	1.95	0.050	2.17	0.030	2.03	0.042	2.08	0.037
		Diversity	2.02	0.045	2.23	0.026	2.03	0.042	2.02	0.043	2.57	0.010
		Turnover	-0.48	0.632	-0.38	0.704	-0.86	0.392	-1.12	0.262	-0.75	0.455
Atlantic terrestrial invertebrates (n = 14, out of 34)	Terrestrial invertebrates	Abundance	-2.66	0.008	-2.38	0.017	-3.15	0.002	-3.07	0.002	-2.90	0.004
		Richness	0.05	0.960	1.56	0.118	0.67	0.500	0.49	0.624	*2.16*	*0.031*
		Diversity	0.51	0.611	1.59	0.112	0.95	0.339	0.36	0.717	1.08	0.281
		Turnover	*2.04*	*0.040*	1.54	0.123	1.10	0.269	1.81	0.070	1.90	0.058
Alpine plants (n = 5, out of 17)	Plants	Abundance	0.39	0.700	0.35	0.727	0.50	0.620	0.51	0.611	0.33	0.734
		Richness	0.28	0.781	0.02	0.983	0.27	0.789	0.10	0.920	0.22	0.822
		Diversity	0.31	0.760	0.24	0.809	0.35	0.724	0.48	0.630	0.23	0.820
		Turnover	2.19	0.029	2.40	0.016	2.43	0.015	2.79	0.005	2.27	0.023
**Random sampling:**	**Model results for BIOGEOREGION:**	**Metric:**	z	p	z	p	z	p	z	p	z	p
Atlantic terrestrial invertebrates (n = 8, out of 34)	Atlantic	Abundance	-2.23	0.026	-2.06	0.040	*-1.65*	*0.099*	-2.37	0.018	*-1.82*	*0.068*
		Richness	-0.02	0.985	-1.21	0.226	-0.56	0.579	-1.22	0.223	-1.12	0.262
		Diversity	0.31	0.753	0.59	0.556	0.90	0.368	0.66	0.507	0.76	0.446
		Turnover	0.01	0.999	0.01	1.000	0.01	0.999	0.01	0.999	0.01	0.999
Alpine plants (n = 6, out of 17)	Alpine	Abundance	-0.89	0.374	-1.50	0.135	-1.06	0.288	-0.98	0.329	-1.54	0.124
		Richness	0.76	0.450	0.99	0.320	1.11	0.267	0.61	0.544	1.19	0.234
		Diversity	-0.86	0.390	-1.20	0.231	-0.71	0.477	-0.96	0.336	-0.80	0.425
		Turnover	3.04	0.002	1.96	0.050	2.23	0.025	2.21	0.027	3.01	0.003

Supplementary Methods:

The following pages report the standardized questionnaire that was filled in by the data providers to deliver information on local anthropogenic pressures at the study sites.

ILTER - biodiversity study

* Required

1. **Contact person:** *

2. **Email:** *

3. **Site:** *

4. **Studied biotic group(s):** *

5. **Ecosystem** *

   Mark only one oval.

   - ◯ Freshwater *After the last question in this section, skip to question 70.*
   - ◯ Marine and transitional zone *After the last question in this section, skip to question 157.*
   - ◯ Terrestrial *After the last question in this section, skip to question 13.*

About the dataset

6. **Is the taxonomic list consistent throughout the whole study period (i.e. same taxonomic resolution during the whole study period, no synonyms)?** *

   Mark only one oval.

   - ◯ Yes
   - ◯ No
   - ◯ Don´t know

7. **Has the taxonomic identification been carried out by the same person or by people with similar expertise during the whole study period?** *

Mark only one oval.

- Yes
- No
- Don´t know

8. **Was the sampling method changed during the study period?** *

Mark only one oval.

- Yes
- No
- Don´t know

9. **Comments:**

10. **Are there neobiota among the surveyed taxa?** *

Mark only one oval.

- Yes
- No
- Don´t know

11. **Are there red-listed species among the surveyed taxa?** *

Mark only one oval.

- Yes
- No
- Don´t know

12. **Would you be able to provide information on these traits for the studied taxa?**

Check all that apply.

- [ ] Maximum organism size
- [ ] Life-cycle duration
- [ ] Number of life cycles per year
- [ ] Life history strategy

Impacts at the site - Terrestrial ecosystems

You are asked to estimate the impact of a series of pressures acting at the site: 1) urban/industrial, 2) agriculture/pasture/forestry, 3) mining/extraction, 4) recreational activities, 5) hunting, 6) artificial elements of landscape (e.g. wind turbines), 7) pollution/nutrients.
You can also indicate whether there are additional pressures or whether major changes occurred at the site during the study period.
At the end of the section you are asked to estimate the overall quality (=naturalness) of the site.

Urban/industrial

13. **Estimate the intensity of urban/industrial impacts at the site:**

If intensity was constant or null throughout the whole study, fill in the first row only. If intensity changed during the study period, identify periods of constant impact (maximum 5 periods) and provide an estimate of the impact in each of the identified periods (rows from 2 to 6)
Mark only one oval per row.

	Null	Weak impact	Medium impact	Strong impact
Constant throughout the whole study	⬭	⬭	⬭	⬭
Non-constant throughout the study: Period 1	⬭	⬭	⬭	⬭
Non-constant throughout the study: Period 2	⬭	⬭	⬭	⬭
Non-constant throughout the study: Period 3	⬭	⬭	⬭	⬭
Non-constant throughout the study: Period 4	⬭	⬭	⬭	⬭
Non-constant throughout the study: Period 5	⬭	⬭	⬭	⬭

14. **If the intensity of impact changed during the study period, indicate the start and end of PERIOD 1:**

year start, year end. E.g.: 1990, 1995

____

15. **If the intensity of impact changed during the study period, indicate the start and end of PERIOD 2:**

year start, year end. E.g.: 1990, 1995

____

16. **If the intensity of impact changed during the study period, indicate the star and end of PERIOD 3:**

year start, year end. E.g.: 1990, 1995

____

17. **If the intensity of impact changed during the study period, indicate the start and end of PERIOD 4:**

year start, year end. E.g.: 1990, 1995

____

18. **If the intensity of impact changed during the study period, indicate the start and end of PERIOD 5:**

year start, year end. E.g.: 1990, 1995

____

19. **Comments**

Agriculture/pasture/forestry

20. **Estimate the intensity of the agriculture/pasture/forestry impact at the site:**
If intensity was constant or null throughout the whole study, fill in the first row only. If intensity changed during the study period, identify periods of constant impact (maximum 5 periods) and provide an estimate of the impact in each of the identified periods (rows from 2 to 6)
Mark only one oval per row.

	Null	Weak impact	Medium impact	Strong impact
Constant throughout the whole study				
Non-constant throughout the study: Period 1				
Non-constant throughout the study: Period 2				
Non-constant throughout the study: Period 3				
Non-constant throughout the study: Period 4				
Non-constant throughout the study: Period 5				

21. **If the intensity of impact changed during the study period, indicate the start and end of PERIOD 1:**
year start, year end. E.g.: 1990, 1995

22. **If the intensity of impact changed during the study period, indicate the start and end of PERIOD 2:**
year start, year end. E.g.: 1990, 1995

23. **If the intensity of impact changed during the study period, indicate the start and end of PERIOD 3:**
year start, year end. E.g.: 1990, 1995

24. **If the intensity of impact changed during the study period, indicate the start and end of PERIOD 4:**
year start, year end. E.g.: 1990, 1995

25. **If the intensity of impact changed during the study period, indicate the start and end of PERIOD 5:**
year start, year end. E.g.: 1990, 1995

26. **Comments**

Mining/extraction

27. **Estimate the intensity of the mining/extraction impact at the site:**
If intensity was constant or null throughout the whole study, fill in the first row only. If intensity changed during the study period, identify periods of constant impact (maximum 5 periods) and provide an estimate of the impact in each of the identified periods (rows from 2 to 6)
Mark only one oval per row.

	Null	Weak impact	Medium impact	Strong impact
Constant throughout the whole study	◯	◯	◯	◯
Non-constant throughout the study: Period 1	◯	◯	◯	◯
Non-constant throughout the study: Period 2	◯	◯	◯	◯
Non-constant throughout the study: Period 3	◯	◯	◯	◯
Non-constant throughout the study: Period 4	◯	◯	◯	◯
Non-constant throughout the study: Period 5	◯	◯	◯	◯

28. **If the intensity of impact changed during the study period, indicate the start and end of PERIOD 1:**
year start, year end. E.g.: 1990, 1995

29. **If the intensity of impact changed during the study period, indicate the start and end of PERIOD 2:**
year start, year end. E.g.: 1990, 1995

30. **If the intensity of impact changed during the study period, indicate the start and end of PERIOD 3:**
year start, year end. E.g.: 1990, 1995

31. **If the intensity of impact changed during the study period, indicate the start and end of PERIOD 4:**
year start, year end. E.g.: 1990, 1995

32. **If the intensity of impact changed during the study period, indicate the start and end of PERIOD 5:**
year start, year end. E.g.: 1990, 1995

33. **Comments**

Recreational activities

34. **Estimate the intensity of recreational activities at the site:**
If intensity was constant or null throughout the whole study, fill in the first row only. If intensity changed during the study period, identify periods of constant impact (maximum 5 periods) and provide an estimate of the impact in each of the identified periods (rows from 2 to 6)
Mark only one oval per row.

	Null	Weak impact	Medium impact	Strong impact
Constant throughout the whole study	◯	◯	◯	◯
Non-constant throughout the study: Period 1	◯	◯	◯	◯
Non-constant throughout the study: Period 2	◯	◯	◯	◯
Non-constant throughout the study: Period 3	◯	◯	◯	◯
Non-constant throughout the study: Period 4	◯	◯	◯	◯
Non-constant throughout the study: Period 5	◯	◯	◯	◯

35. **If the intensity of impact changed during the study period, indicate the start and end of PERIOD 1:**
year start, year end. E.g.: 1990, 1995

36. **If the intensity of impact changed during the study period, indicate the start and end of PERIOD 2:**
year start, year end. E.g.: 1990, 1995

37. **If the intensity of impact changed during the study period, indicate the start and end of PERIOD 3:**
year start, year end. E.g.: 1990, 1995

38. **If the intensity of impact changed during the study period, indicate the start and end of PERIOD 4:**
year start, year end. E.g.: 1990, 1995

39. **If the intensity of impact changed during the study period, indicate the start and end of PERIOD 5:**
year start, year end. E.g.: 1990, 1995

40. **Comments**

Hunting

41. **Estimate the intensity of the impact of hunting at the site:**
If intensity was constant or null throughout the whole study, fill in the first row only. If intensity changed during the study period, identify periods of constant impact (maximum 5 periods) and provide an estimate of the impact in each of the identified periods (rows from 2 to 6)
Mark only one oval per row.

	Null	Weak impact	Medium impact	Strong impact
Constant throughout the whole study	◯	◯	◯	◯
Non-constant throughout the study: Period 1	◯	◯	◯	◯
Non-constant throughout the study: Period 2	◯	◯	◯	◯
Non-constant throughout the study: Period 3	◯	◯	◯	◯
Non-constant throughout the study: Period 4	◯	◯	◯	◯
Non-constant throughout the study: Period 5	◯	◯	◯	◯

42. **If the intensity of impact changed during the study period, indicate the start and end of PERIOD 2:**
year start, year end. E.g.: 1990, 1995

43. **If the intensity of impact changed during the study period, indicate the start and end of PERIOD 3:**
year start, year end. E.g.: 1990, 1995

44. **If the intensity of impact changed during the study period, indicate the start and end of PERIOD 4:**
year start, year end. E.g.: 1990, 1995

45. **If the intensity of impact changed during the study period, indicate the start and end of PERIOD 5:**
year start, year end. E.g.: 1990, 1995

46. **Comments**

Artificial elements of landscape (e.g. wind turbines)

47. **Estimate the intensity of the impact of artificial elements of landscape at the site:**
If intensity was constant or null throughout the whole study, fill in the first row only. If intensity changed during the study period, identify periods of constant impact (maximum 5 periods) and provide an estimate of the impact in each of the identified periods (rows from 2 to 6)
Mark only one oval per row.

	Null	Weak impact	Medium impact	Strong impact
Constant throughout the whole study				
Non-constant throughout the study: Period 1				
Non-constant throughout the study: Period 2				
Non-constant throughout the study: Period 3				
Non-constant throughout the study: Period 4				
Non-constant throughout the study: Period 5				

48. **If the intensity of impact changed during the study period, indicate the start and end of PERIOD 1:**
year start, year end. E.g.: 1990, 1995

49. **If the intensity of impact changed during the study period, indicate the start and end of PERIOD 2:**

year start, year end. E.g.: 1990, 1995

50. **If the intensity of impact changed during the study period, indicate the start and end of PERIOD 3:**

year start, year end. E.g.: 1990, 1995

51. **If the intensity of impact changed during the study period, indicate the start and end of PERIOD 4:**

year start, year end. E.g.: 1990, 1995

52. **If the intensity of impact changed during the study period, indicate the start and end of PERIOD 5:**

year start, year end. E.g.: 1990, 1995

53. **Comments**

Sources of pollution/nutrients

54. **Estimate the intensity of the impact of pollution/nutrients at the site:**

If intensity was constant or null throughout the whole study, fill in the first row only. If intensity changed during the study period, identify periods of constant impact (maximum 5 periods) and provide an estimate of the impact in each of the identified periods (rows from 2 to 6)

Mark only one oval per row.

	Null	Weak impact	Medium impact	Strong impact
Constant throughout the whole study	⬭	⬭	⬭	⬭
Non-constant throughout the study: Period 1	⬭	⬭	⬭	⬭
Non-constant throughout the study: Period 2	⬭	⬭	⬭	⬭
Non-constant throughout the study: Period 3	⬭	⬭	⬭	⬭
Non-constant throughout the study: Period 4	⬭	⬭	⬭	⬭
Non-constant throughout the study: Period 5	⬭	⬭	⬭	⬭

55. **If the intensity of impact changed during the study period, indicate the start and end of PERIOD 1:**

year start, year end. E.g.: 1990, 1995

56. **If the intensity of impact changed during the study period, indicate the start and end of PERIOD 2:**

year start, year end. E.g.: 1990, 1995

57. **If the intensity of impact changed during the study period, indicate the start and end of PERIOD 3:**

year start, year end. E.g.: 1990, 1995

58. **If the intensity of impact changed during the study period, indicate the start and end of PERIOD 4:**

year start, year end. E.g.: 1990, 1995

59. **If the intensity of impact changed during the study period, indicate the start and end of PERIOD 5:**

year start, year end. E.g.: 1990, 1995

60. **Comments**

Additional pressures and major changes at the site

61. **Did other major changes occur at the site and its surroundings that could have affected the biodiversity trends during the study period? (e.g. additional pressures, changes in nutrient inputs/deposition, changes in levels of acidification, parasite/desease outbreaks, changed pressure by predators, increased/decreased availability of resources, etc...)** *

Mark only one oval.

- Yes
- No
- Don´t know

62. **If yes, explain WHAT happened and WHEN**

Overall site assessment

63. **Estimate the quality (=naturalness) of the site:**

If quality was constant throughout the whole study, fill in the first row only. If quality changed during the study period, identify periods of constant quality (maximum 5 periods) and provide an estimate of the quality in each of the identified periods (rows from 2 to 6)

Mark only one oval per row.

	Bad	Poor	Moderate	Good	High
Constant throughout the whole study					
Non-constant throughout the study: Period 1					
Non-constant throughout the study: Period 2					
Non-constant throughout the study: Period 3					
Non-constant throughout the study: Period 4					
Non-constant throughout the study: Period 5					

64. **If the quality changed during the study period, indicate the start and end of PERIOD 1:**

year start, year end. E.g.: 1990, 1995

65. **If quality changed during the study period, indicate the start and end of PERIOD 2:**

year start, year end. E.g.: 1990, 1995

66. **If quality changed during the study period, indicate the start and end of PERIOD 3:**

year start, year end. E.g.: 1990, 1995

67. **If quality changed during the study period, indicate the start and end of PERIOD 4:**

year start, year end. E.g.: 1990, 1995

68. **If quality changed during the study period, indicate the start and end of PERIOD 5:**
year start, year end. E.g.: 1990, 1995

69. **Comments**

Thank you!

Stop filling out this form.

Impacts at the site - Freshwater ecosystems

You are asked to estimate the impact of a series of pressures acting at the site: 1) urban/industrial, 2) agriculture/pasture/forestry, 3) aquaculture, 4) mining/extraction, 5) recreational activities, 6) fishing/hunting, 7) hydrological alterations , 8) morphological alterations of the aquatic environment, 9) morphological alterations of the riparian/costal environment, 10) harbours/marinas/navigation, 11) pollution/nutrients/warm water
You can also indicate whether there are additional pressures or whether major changes occurred at the site during the study period.
At the end of the section you are asked to estimate the overall quality (=naturalness) of the site.

Urban/industrial

70. **Estimate the intensity of the urban/industrial impact at the site:**
If intensity was constant or null throughout the whole study, fill in the first row only. If intensity changed during the study period, identify periods of constant impact (maximum 5 periods) and provide an estimate of the impact in each of the identified periods (rows from 2 to 6)
Mark only one oval per row.

	Null	Weak impact	Medium impact	Strong impact
Constant throughout the whole study	◯	◯	◯	◯
Non-constant throughout the study: Period 1	◯	◯	◯	◯
Non-constant throughout the study: Period 2	◯	◯	◯	◯
Non-constant throughout the study: Period 3	◯	◯	◯	◯
Non-constant throughout the study: Period 4	◯	◯	◯	◯
Non-constant throughout the study: Period 5	◯	◯	◯	◯

71. **If the intensity of impact changed during the study period, indicate the start and end of PERIOD 1:**
year start, year end. E.g.: 1990, 1995

72. **If the intensity of impact changed during the study period, indicate the start and end of PERIOD 2:**

year start, year end. E.g.: 1990, 1995

73. **If the intensity of impact changed during the study period, indicate the start and end of PERIOD 3:**

year start, year end. E.g.: 1990, 1995

74. **If the intensity of impact changed during the study period, indicate the start and end of PERIOD 4:**

year start, year end. E.g.: 1990, 1995

75. **If the intensity of impact changed during the study period, indicate the start and end of PERIOD 5:**

year start, year end. E.g.: 1990, 1995

76. **Comments**

Agriculture/pasture/forestry

77. **Estimate the intensity of the agriculture/pasture/forestry impact at the site:**

If intensity was constant or null throughout the whole study, fill in the first row only. If intensity changed during the study period, identify periods of constant impact (maximum 5 periods) and provide an estimate of the impact in each of the identified periods (rows from 2 to 6)

Mark only one oval per row.

	Null	Weak impact	Medium impact	Strong impact
Constant throughout the whole study	◯	◯	◯	◯
Non-constant throughout the study: Period 1	◯	◯	◯	◯
Non-constant throughout the study: Period 2	◯	◯	◯	◯
Non-constant throughout the study: Period 3	◯	◯	◯	◯
Non-constant throughout the study: Period 4	◯	◯	◯	◯
Non-constant throughout the study: Period 5	◯	◯	◯	◯

78. **If the intensity of impact changed during the study period, indicate the start and end of PERIOD 1:**

year start, year end. E.g.: 1990, 1995

79. **If the intensity of impact changed during the study period, indicate the start and end of PERIOD 2:**

year start, year end. E.g.: 1990, 1995

80. **If the intensity of impact changed during the study period, indicate the start and end of PERIOD 3:**

year start, year end. E.g.: 1990, 1995

81. **If the intensity of impact changed during the study period, indicate the start and end of PERIOD 4:**

year start, year end. E.g.: 1990, 1995

82. **If the intensity of impact changed during the study period, indicate the start and end of PERIOD 5:**

year start, year end. E.g.: 1990, 1995

83. **Comments**

Aquaculture

84. **Estimate the intensity of the impact of aquaculture at the site:**

If intensity was constant or null throughout the whole study, fill in the first row only. If intensity changed during the study period, identify periods of constant impact (maximum 5 periods) and provide an estimate of the impact in each of the identified periods (rows from 2 to 6)

Mark only one oval per row.

	Null	Weak impact	Medium impact	Strong impact
Constant throughout the whole study	⬭	⬭	⬭	⬭
Non-constant throughout the study: Period 1	⬭	⬭	⬭	⬭
Non-constant throughout the study: Period 2	⬭	⬭	⬭	⬭
Non-constant throughout the study: Period 3	⬭	⬭	⬭	⬭
Non-constant throughout the study: Period 4	⬭	⬭	⬭	⬭
Non-constant throughout the study: Period 5	⬭	⬭	⬭	⬭

85. **If the intensity of impact changed during the study period, indicate the start and end of PERIOD 1:**

year start, year end. E.g.: 1990, 1995

86. **If the intensity of impact changed during the study period, indicate the start and end of PERIOD 2:**

year start, year end. E.g.: 1990, 1995

87. **If the intensity of impact changed during the study period, indicate the start and end of PERIOD 3:**

year start, year end. E.g.: 1990, 1995

88. **If the intensity of impact changed during the study period, indicate the start and end of PERIOD 4:**

year start, year end. E.g.: 1990, 1995

89. **If the intensity of impact changed during the study period, indicate the start and end of PERIOD 5:**

year start, year end. E.g.: 1990, 1995

90. **Comments**

Mining/extraction

91. **Estimate the intensity of the mining/extraction impact at the site:**
If intensity was constant or null throughout the whole study, fill in the first row only. If intensity changed during the study period, identify periods of constant impact (maximum 5 periods) and provide an estimate of the impact in each of the identified periods (rows from 2 to 6)
Mark only one oval per row.

	Null	Weak impact	Medium impact	Strong impact
Constant throughout the whole study				
Non-constant throughout the study: Period 1				
Non-constant throughout the study: Period 2				
Non-constant throughout the study: Period 3				
Non-constant throughout the study: Period 4				
Non-constant throughout the study: Period 5				

92. **If the intensity of impact changed during the study period, indicate the start and end of PERIOD 1:**
year start, year end. E.g.: 1990, 1995

93. **If the intensity of impact changed during the study period, indicate the start and end of PERIOD 2:**
year start, year end. E.g.: 1990, 1995

94. **If the intensity of impact changed during the study period, indicate the start and end of PERIOD 3:**
year start, year end. E.g.: 1990, 1995

95. **If the intensity of impact changed during the study period, indicate the start and end of PERIOD 4**
year start, year end. E.g.: 1990, 1995

96. **If the intensity of impact changed during the study period, indicate the start and end of PERIOD 5:**

year start, year end. E.g.: 1990, 1995

97. **Comments**

Recreational activities

98. **Estimate the intensity of the impact of recreational activities at the site:**

If intensity was constant or null throughout the whole study, fill in the first row only. If intensity changed during the study period, identify periods of constant impact (maximum 5 periods) and provide an estimate of the impact in each of the identified periods (rows from 2 to 6)
Mark only one oval per row.

	Null	Weak impact	Medium impact	Strong impact
Constant throughout the whole study	◯	◯	◯	◯
Non-constant throughout the study: Period 1	◯	◯	◯	◯
Non-constant throughout the study: Period 2	◯	◯	◯	◯
Non-constant throughout the study: Period 3	◯	◯	◯	◯
Non-constant throughout the study: Period 4	◯	◯	◯	◯
Non-constant throughout the study: Period 5	◯	◯	◯	◯

99. **If the intensity of impact changed during the study period, indicate the start and end of PERIOD 1:**

year start, year end. E.g.: 1990, 1995

100. **If the intensity of impact changed during the study period, indicate the start and end of PERIOD 2:**

year start, year end. E.g.: 1990, 1995

101. **If the intensity of impact changed during the study period, indicate the start and end of PERIOD 3:**

year start, year end. E.g.: 1990, 1995

102. **If the intensity of impact changed during the study period, indicate the start and end of PERIOD 4:**

year start, year end. E.g.: 1990, 1995

103. **If the intensity of impact changed during the study period, indicate the start and end of PERIOD 5:**

year start, year end. E.g.: 1990, 1995

104. **Comments**

Fishing/hunting

105. **Estimate the intensity of the impact of fishing/hunting at the site:**

If intensity was constant or null throughout the whole study, fill in the first row only. If intensity changed during the study period, identify periods of constant impact (maximum 5 periods) and provide an estimate of the impact in each of the identified periods (rows from 2 to 6)
Mark only one oval per row.

	Null	Weak impact	Medium impact	Strong impact
Constant throughout the whole study	◯	◯	◯	◯
Non-constant throughout the study: Period 1	◯	◯	◯	◯
Non-constant throughout the study: Period 2	◯	◯	◯	◯
Non-constant throughout the study: Period 3	◯	◯	◯	◯
Non-constant throughout the study: Period 4	◯	◯	◯	◯
Non-constant throughout the study: Period 5	◯	◯	◯	◯

106. **If the intensity of impact changed during the study period, indicate the start and end of PERIOD 1:**

year start, year end. E.g.: 1990, 1995

107. **If the intensity of impact changed during the study period, indicate the start and end of PERIOD 2:**

year start, year end. E.g.: 1990, 1995

108. **If the intensity of impact changed during the study period, indicate the start and end of PERIOD 3:**

year start, year end. E.g.: 1990, 1995

109. **If the intensity of impact changed during the study period, indicate the start and end of PERIOD 4:**

year start, year end. E.g.: 1990, 1995

110. **If the intensity of impact changed during the study period, indicate the start and end of PERIOD 5:**

year start, year end. E.g.: 1990, 1995

111. **Comments**

Hydrological alterations (e.g. water abstraction, dam operations)

112. **Estimate the intensity of the impact of hydrological alterations at the site:**

If intensity was constant or null throughout the whole study, fill in the first row only. If intensity changed during the study period, identify periods of constant impact (maximum 5 periods) and provide an estimate of the impact in each of the identified periods (rows from 2 to 6)
Mark only one oval per row.

	Null	Weak impact	Medium impact	Strong impact
Constant throughout the whole study	⬭	⬭	⬭	⬭
Non-constant throughout the study: Period 1	⬭	⬭	⬭	⬭
Non-constant throughout the study: Period 2	⬭	⬭	⬭	⬭
Non-constant throughout the study: Period 3	⬭	⬭	⬭	⬭
Non-constant throughout the study: Period 4	⬭	⬭	⬭	⬭
Non-constant throughout the study: Period 5	⬭	⬭	⬭	⬭

113. **If the intensity of impact changed during the study period, indicate the start and end of PERIOD 1:**

year start, year end. E.g.: 1990, 1995

114. **If the intensity of impact changed during the study period, indicate the start and end of PERIOD 2:**

year start, year end. E.g.: 1990, 1995

115. **If the intensity of impact changed during the study period, indicate the start and end of PERIOD 3:**

year start, year end. E.g.: 1990, 1995

116. **If the intensity of impact changed during the study period, indicate the start and end of PERIOD 4:**

year start, year end. E.g.: 1990, 1995

117. **If the intensity of impact changed during the study period, indicate the start and end of PERIOD 5:**

year start, year end. E.g.: 1990, 1995

118. **Comments**

Morphological alterations of the aquatic environment (e.g. embankment, channelization, installation of underwater structures such as pillars, ripraps, flow deflectors, removal of natural underwater structures such as wood logs, boulders etc...)

119. **Estimate the intensity of the impact of morphological alterations of the aquatic environment at the site:**

If intensity was constant or null throughout the whole study, fill in the first row only. If intensity changed during the study period, identify periods of constant impact (maximum 5 periods) and provide an estimate of the impact in each of the identified periods (rows from 2 to 6)
Mark only one oval per row.

	Null	Weak impact	Medium impact	Strong impact
Constant throughout the whole study	⬭	⬭	⬭	⬭
Non-constant throughout the study: Period 1	⬭	⬭	⬭	⬭
Non-constant throughout the study: Period 2	⬭	⬭	⬭	⬭
Non-constant throughout the study: Period 3	⬭	⬭	⬭	⬭
Non-constant throughout the study: Period 4	⬭	⬭	⬭	⬭
Non-constant throughout the study: Period 5	⬭	⬭	⬭	⬭

120. **If the intensity of impact changed during the study period, indicate the start and end of PERIOD 1:**

year start, year end. E.g.: 1990, 1995

____

121. **If the intensity of impact changed during the study period, indicate the start and end of PERIOD 2:**

year start, year end. E.g.: 1990, 1995

____

122. **If the intensity of impact changed during the study period, indicate the start and end of PERIOD 3:**

year start, year end. E.g.: 1990, 1995

____

123. **If the intensity of impact changed during the study period, indicate the start and end of PERIOD 4:**

year start, year end. E.g.: 1990, 1995

____

124. **If the intensity of impact changed during the study period, indicate the start and end of PERIOD 5:**

year start, year end. E.g.: 1990, 1995

____

125. **Comments**

Morphological alterations of the riparian/costal environment (e.g. removal of riparian/fringing vegetation)

126. **Estimate the intensity of the impact of morphological alterations of the riparian/costal environment at the site:**

If intensity was constant or null throughout the whole study, fill in the first row only. If intensity changed during the study period, identify periods of constant impact (maximum 5 periods) and provide an estimate of the impact in each of the identified periods (rows from 2 to 6)

Mark only one oval per row.

	Null	Weak impact	Medium impact	Strong impact
Constant throughout the whole study				
Non-constant throughout the study: Period 1				
Non-constant throughout the study: Period 2				
Non-constant throughout the study: Period 3				
Non-constant throughout the study: Period 4				
Non-constant throughout the study: Period 5				

127. **If the intensity of impact changed during the study period, indicate the start and end of PERIOD 1:**

year start, year end. E.g.: 1990, 1995

128. **If the intensity of impact changed during the study period, indicate the start and end of PERIOD 2:**

year start, year end. E.g.: 1990, 1995

129. **If the intensity of impact changed during the study period, indicate the start and end of PERIOD 3:**

year start, year end. E.g.: 1990, 1995

130. **If the intensity of impact changed during the study period, indicate the start and end of PERIOD 4:**

year start, year end. E.g.: 1990, 1995

131. **If the intensity of impact changed during the study period, indicate the start and end of PERIOD 5:**
year start, year end. E.g.: 1990, 1995

132. **Comments**

Harbours/marinas/navigation

133. **Estimate the intensity of the impact of harbours/marinas/navigation at the site:**
If intensity was constant or null throughout the whole study, fill in the first row only. If intensity changed during the study period, identify periods of constant impact (maximum 5 periods) and provide an estimate of the impact in each of the identified periods (rows from 2 to 6)
Mark only one oval per row.

	Null	Weak impact	Medium impact	Strong impact
Constant throughout the whole study	⬭	⬭	⬭	⬭
Non-constant throughout the study: Period 1	⬭	⬭	⬭	⬭
Non-constant throughout the study: Period 2	⬭	⬭	⬭	⬭
Non-constant throughout the study: Period 3	⬭	⬭	⬭	⬭
Non-constant throughout the study: Period 4	⬭	⬭	⬭	⬭
Non-constant throughout the study: Period 5	⬭	⬭	⬭	⬭

134. **If the intensity of impact changed during the study period, indicate the start and end of PERIOD 1:**
year start, year end. E.g.: 1990, 1995

135. **If the intensity of impact changed during the study period, indicate the start and end of PERIOD 2:**
year start, year end. E.g.: 1990, 1995

136. **If the intensity of impact changed during the study period, indicate the start and end of PERIOD 3:**
year start, year end. E.g.: 1990, 1995

137. **If the intensity of impact changed during the study period, indicate the start and end of PERIOD 4:**

year start, year end. E.g.: 1990, 1995

138. **If the intensity of impact changed during the study period, indicate the start and end of PERIOD 5:**

year start, year end. E.g.: 1990, 1995

139. **Comments**

Sources of pollution/nutrients/warm water (e.g. from industrial sites)

140. **Estimate the intensity of the impact of pollution/nutrients/warm water at the site:**

If intensity was constant or null throughout the whole study, fill in the first row only. If intensity changed during the study period, identify periods of constant impact (maximum 5 periods) and provide an estimate of the impact in each of the identified periods (rows from 2 to 6)

Mark only one oval per row.

	Null	Weak impact	Medium impact	Strong impact
Constant throughout the whole study				
Non-constant throughout the study: Period 1				
Non-constant throughout the study: Period 2				
Non-constant throughout the study: Period 3				
Non-constant throughout the study: Period 4				
Non-constant throughout the study: Period 5				

141. **If the intensity of impact changed during the study period, indicate the start and end of PERIOD 1:**

year start, year end. E.g.: 1990, 1995

142. **If the intensity of impact changed during the study period, indicate the start and end of PERIOD 2:**

year start, year end. E.g.: 1990, 1995

143. **If the intensity of impact changed during the study period, indicate the start and end of PERIOD 3:**

year start, year end. E.g.: 1990, 1995

144. **If the intensity of impact changed during the study period, indicate the start and end of PERIOD 4:**

year start, year end. E.g.: 1990, 1995

145. **If the intensity of impact changed during the study period, indicate the start and end of PERIOD 5:**

year start, year end. E.g.: 1990, 1995

146. **Comments**

Additional pressures and major changes at the site

147. **Did other major changes occur at the site and its surroundings that could have affected the biodiversity trends during the study period? (e.g. additional pressures, changes in nutrient inputs/deposition, changes in levels of acidification, parasite/desease outbreaks, changed pressure by predators, increased/decreased availability of resources, etc...)** *

Mark only one oval.

- Yes
- No
- Don´t know

148. **If yes, explain WHAT happened and WHEN**

Overall site assessment

149. **Estimate the quality (=naturalness) of the site:**

If quality was constant throughout the whole study, fill in the first row only. If quality changed during the study period, identify periods of constant quality (maximum 5) and provide an estimate of the quality in each of the identified periods (rows from 2 to 6)

Mark only one oval per row.

	Bad	Poor	Moderate	Good	High
Constant throughout the whole study	⬭	⬭	⬭	⬭	⬭
Non-constant throughout the study: Period 1	⬭	⬭	⬭	⬭	⬭
Non-constant throughout the study: Period 2	⬭	⬭	⬭	⬭	⬭
Non-constant throughout the study: Period 3	⬭	⬭	⬭	⬭	⬭
Non-constant throughout the study: Period 4	⬭	⬭	⬭	⬭	⬭
Non-constant throughout the study: Period 5	⬭	⬭	⬭	⬭	⬭

150. **If quality changed during the study period, indicate the start and end of PERIOD 1:**

year start, year end. E.g.: 1990, 1995

____

151. **If quality changed during the study period, indicate the start and end of PERIOD 2:**

year start, year end. E.g.: 1990, 1995

____

152. **If quality changed during the study period, indicate the start and end of PERIOD 3:**

year start, year end. E.g.: 1990, 1995

____

153. **If quality changed during the study period, indicate the start and end of PERIOD 4:**

year start, year end. E.g.: 1990, 1995

____

154. **If quality changed during the study period, indicate the start and end of PERIOD 5:**

year start, year end. E.g.: 1990, 1995

____

155. **Comments**

____

____

____

____

____

Data availability - Water temperature

156. **Are water temperature data available for the whole study period?**

Mark only one oval.

- Yes
- No

Thank you!

Stop filling out this form.

Impacts at the site - Marine and transitional ecosystems

You are asked to estimate the impact of a series of pressures acting at the site: 1) urban/industrial, 2) agriculture/pasture/forestry, 3) aquaculture, 4) mining/extraction, 5) recreational activities, 6) fishing/hunting, 7) offshore drilling , 8) morphological alterations of the aquatic environment, 9) morphological alterations of the riparian/costal environment, 10) harbours/marinas/navigation, 11) pollution/nutrients/warm water
You can also indicate whether there are additional pressures or whether major changes occurred at the site during the study period.
At the end of the section you are asked to estimate the overall quality (=naturalness) of the site.

Urban/industrial

157. **Estimate the intensity of the urban/industrial impact at the site:**

If intensity was constant or null throughout the whole study, fill in the first row only. If intensity changed during the study period, identify periods of constant impact (maximum 5 periods) and provide an estimate of the impact in each of the identified periods (rows from 2 to 6)
Mark only one oval per row.

	Null	Weak impact	Medium impact	Strong impact
Constant throughout the whole study				
Non-constant throughout the study: Period 1				
Non-constant throughout the study: Period 2				
Non-constant throughout the study: Period 3				
Non-constant throughout the study: Period 4				
Non-constant throughout the study: Period 5				

158. **If the intensity of impact changed during the study period, indicate the start and end of PERIOD 1:**

year start, year end. E.g.: 1990, 1995

159. **If the intensity of impact changed during the study period, indicate the start and end of PERIOD 2:**

year start, year end. E.g.: 1990, 1995

160. **If the intensity of impact changed during the study period, indicate the start and end of PERIOD 3:**

year start, year end. E.g.: 1990, 1995

161. **If the intensity of impact changed during the study period, indicate the start and end of PERIOD 4:**

year start, year end. E.g.: 1990, 1995

162. **If the intensity of impact changed during the study period, indicate the start and end of PERIOD 5:**

year start, year end. E.g.: 1990, 1995

163. **Comments**

Agriculture/pasture/forestry

164. **Estimate the intensity of the agriculture/pasture/forestry impact at the site:**

If intensity was constant or null throughout the whole study, fill in the first row only. If intensity changed during the study period, identify periods of constant impact (maximum 5 periods) and provide an estimate of the impact in each of the identified periods (rows from 2 to 6)
Mark only one oval per row.

	Null	Weak impact	Medium impact	Strong impact
Constant throughout the whole study	◯	◯	◯	◯
Non-constant throughout the study: Period 1	◯	◯	◯	◯
Non-constant throughout the study: Period 2	◯	◯	◯	◯
Non-constant throughout the study: Period 3	◯	◯	◯	◯
Non-constant throughout the study: Period 4	◯	◯	◯	◯
Non-constant throughout the study: Period 5	◯	◯	◯	◯

165. **If the intensity of impact changed during the study period, indicate the start and end of PERIOD 1:**

year start, year end. E.g.: 1990, 1995

166. **If the intensity of impact changed during the study period, indicate the start and end of PERIOD 2:**

year start, year end. E.g.: 1990, 1995

167. **If the intensity of impact changed during the study period, indicate the start and end of PERIOD 3:**

year start, year end. E.g.: 1990, 1995

168. **If the intensity of impact changed during the study period, indicate the start and end of PERIOD 4:**

year start, year end. E.g.: 1990, 1995

169. **If the intensity of impact changed during the study period, indicate the start and end of PERIOD 5:**

year start, year end. E.g.: 1990, 1995

170. **Comments**

Aquaculture

171. **Estimate the intensity of the impact of aquaculture at the site:**

If intensity was constant or null throughout the whole study, fill in the first row only. If intensity changed during the study period, identify periods of constant impact (maximum 5 periods) and provide an estimate of the impact in each of the identified periods (rows from 2 to 6)

Mark only one oval per row.

	Null	Weak impact	Medium impact	Strong impact
Constant throughout the whole study	◯	◯	◯	◯
Non-constant throughout the study: Period 1	◯	◯	◯	◯
Non-constant throughout the study: Period 2	◯	◯	◯	◯
Non-constant throughout the study: Period 3	◯	◯	◯	◯
Non-constant throughout the study: Period 4	◯	◯	◯	◯
Non-constant throughout the study: Period 5	◯	◯	◯	◯

172. **If the intensity of impact changed during the study period, indicate the start and end of PERIOD 1:**
year start, year end. E.g.: 1990, 1995

173. **If the intensity of impact changed during the study period, indicate the start and end of PERIOD 2:**
year start, year end. E.g.: 1990, 1995

174. **If the intensity of impact changed during the study period, indicate the start and end of PERIOD 3:**
year start, year end. E.g.: 1990, 1995

175. **If the intensity of impact changed during the study period, indicate the start and end of PERIOD 4:**
year start, year end. E.g.: 1990, 1995

176. **If the intensity of impact changed during the study period, indicate the start and end of PERIOD 5:**
year start, year end. E.g.: 1990, 1995

177. **Comments**

Mining/extraction

178. **Estimate the intensity of the mining/extraction impact at the site:**

If intensity was constant or null throughout the whole study, fill in the first row only. If intensity changed during the study period, identify periods of constant impact (maximum 5 periods) and provide an estimate of the impact in each of the identified periods (rows from 2 to 6)

Mark only one oval per row.

	Null	Weak impact	Medium impact	Strong impact
Constant throughout the whole study	⬭	⬭	⬭	⬭
Non-constant throughout the study: Period 1	⬭	⬭	⬭	⬭
Non-constant throughout the study: Period 2	⬭	⬭	⬭	⬭
Non-constant throughout the study: Period 3	⬭	⬭	⬭	⬭
Non-constant throughout the study: Period 4	⬭	⬭	⬭	⬭
Non-constant throughout the study: Period 5	⬭	⬭	⬭	⬭

179. **If the intensity of impact changed during the study period, indicate the start and end of PERIOD 1:**

year start, year end. E.g.: 1990, 1995

____

180. **If the intensity of impact changed during the study period, indicate the start and end of PERIOD 2:**

year start, year end. E.g.: 1990, 1995

____

181. **If the intensity of impact changed during the study period, indicate the start and end of PERIOD 3:**

year start, year end. E.g.: 1990, 1995

____

182. **If the intensity of impact changed during the study period, indicate the start and end of PERIOD 4**

year start, year end. E.g.: 1990, 1995

____

183. **If the intensity of impact changed during the study period, indicate the start and end of PERIOD 5:**

year start, year end. E.g.: 1990, 1995

____

184. **Comments**

____

____

____

____

____

Recreational activities

185. **Estimate the intensity of the impact of recreational activities at the site:**
If intensity was constant or null throughout the whole study, fill in the first row only. If intensity changed during the study period, identify periods of constant impact (maximum 5 periods) and provide an estimate of the impact in each of the identified periods (rows from 2 to 6)
Mark only one oval per row.

	Null	Weak impact	Medium impact	Strong impact
Constant throughout the whole study	◯	◯	◯	◯
Non-constant throughout the study: Period 1	◯	◯	◯	◯
Non-constant throughout the study: Period 2	◯	◯	◯	◯
Non-constant throughout the study: Period 3	◯	◯	◯	◯
Non-constant throughout the study: Period 4	◯	◯	◯	◯
Non-constant throughout the study: Period 5	◯	◯	◯	◯

186. **If the intensity of impact changed during the study period, indicate the start and end of PERIOD 1:**
year start, year end. E.g.: 1990, 1995

____

187. **If the intensity of impact changed during the study period, indicate the start and end of PERIOD 2:**
year start, year end. E.g.: 1990, 1995

____

188. **If the intensity of impact changed during the study period, indicate the start and end of PERIOD 3:**
year start, year end. E.g.: 1990, 1995

____

189. **If the intensity of impact changed during the study period, indicate the start and end of PERIOD 4:**
year start, year end. E.g.: 1990, 1995

____

190. **If the intensity of impact changed during the study period, indicate the start and end of PERIOD 5:**
year start, year end. E.g.: 1990, 1995

191. **Comments**

Fishing/hunting

192. **Estimate the intensity of the impact of fishing/hunting at the site:**
If intensity was constant or null throughout the whole study, fill in the first row only. If intensity changed during the study period, identify periods of constant impact (maximum 5 periods) and provide an estimate of the impact in each of the identified periods (rows from 2 to 6)
Mark only one oval per row.

	Null	Weak impact	Medium impact	Strong impact
Constant throughout the whole study				
Non-constant throughout the study: Period 1				
Non-constant throughout the study: Period 2				
Non-constant throughout the study: Period 3				
Non-constant throughout the study: Period 4				
Non-constant throughout the study: Period 5				

193. **If the intensity of impact changed during the study period, indicate the start and end of PERIOD 1:**
year start, year end. E.g.: 1990, 1995

194. **If the intensity of impact changed during the study period, indicate the start and end of PERIOD 2:**
year start, year end. E.g.: 1990, 1995

195. **If the intensity of impact changed during the study period, indicate the start and end of PERIOD 3:**
year start, year end. E.g.: 1990, 1995

196. **If the intensity of impact changed during the study period, indicate the start and end of PERIOD 4:**

year start, year end. E.g.: 1990, 1995

197. **If the intensity of impact changed during the study period, indicate the start and end of PERIOD 5:**

year start, year end. E.g.: 1990, 1995

198. **Comments**

Offshore drilling

199. **Estimate the intensity of the impact of offshore drilling at the site:**

If intensity was constant or null throughout the whole study, fill in the first row only. If intensity changed during the study period, identify periods of constant impact (maximum 5 periods) and provide an estimate of the impact in each of the identified periods (rows from 2 to 6)
Mark only one oval per row.

	Null	Weak impact	Medium impact	Strong impact
Constant throughout the whole study	⬭	⬭	⬭	⬭
Non-constant throughout the study: Period 1	⬭	⬭	⬭	⬭
Non-constant throughout the study: Period 2	⬭	⬭	⬭	⬭
Non-constant throughout the study: Period 3	⬭	⬭	⬭	⬭
Non-constant throughout the study: Period 4	⬭	⬭	⬭	⬭
Non-constant throughout the study: Period 5	⬭	⬭	⬭	⬭

200. **If the intensity of impact changed during the study period, indicate the start and end of PERIOD 1:**

year start, year end. E.g.: 1990, 1995

201. **If the intensity of impact changed during the study period, indicate the start and end of PERIOD 2:**

year start, year end. E.g.: 1990, 1995

202. **If the intensity of impact changed during the study period, indicate the start and end of PERIOD 3:**

year start, year end. E.g.: 1990, 1995

203. **If the intensity of impact changed during the study period, indicate the start and end of PERIOD 4:**

year start, year end. E.g.: 1990, 1995

204. **If the intensity of impact changed during the study period, indicate the start and end of PERIOD 5:**

year start, year end. E.g.: 1990, 1995

205. **Comments**

Morphological alterations of the aquatic environment (e.g. embankment, channelization, installation of underwater structures such as pillars, ripraps, flow deflectors, removal of natural underwater structures such as wood logs, boulders etc...)

206. **Estimate the intensity of the impact of morphological alterations of the aquatic environment at the site:**

If intensity was constant or null throughout the whole study, fill in the first row only. If intensity changed during the study period, identify periods of constant impact (maximum 5 periods) and provide an estimate of the impact in each of the identified periods (rows from 2 to 6)
Mark only one oval per row.

	Null	Weak impact	Medium impact	Strong impact
Constant throughout the whole study				
Non-constant throughout the study: Period 1				
Non-constant throughout the study: Period 2				
Non-constant throughout the study: Period 3				
Non-constant throughout the study: Period 4				
Non-constant throughout the study: Period 5				

207. **If the intensity of impact changed during the study period, indicate the start and end of PERIOD 1:**

year start, year end. E.g.: 1990, 1995

208. **If the intensity of impact changed during the study period, indicate the start and end of PERIOD 2:**

year start, year end. E.g.: 1990, 1995

209. **If the intensity of impact changed during the study period, indicate the start and end of PERIOD 3:**

year start, year end. E.g.: 1990, 1995

210. **If the intensity of impact changed during the study period, indicate the start and end of PERIOD 4:**

year start, year end. E.g.: 1990, 1995

211. **If the intensity of impact changed during the study period, indicate the start and end of PERIOD 5:**

year start, year end. E.g.: 1990, 1995

212. **Comments**

Morphological alterations of the riparian/costal environment (e.g. removal of riparian/fringing vegetation)

213. **Estimate the intensity of the impact of morphological alterations of the riparian/costal environment at the site:**

If intensity was constant or null throughout the whole study, fill in the first row only. If intensity changed during the study period, identify periods of constant impact (maximum 5 periods) and provide an estimate of the impact in each of the identified periods (rows from 2 to 6)
Mark only one oval per row.

	Null	Weak impact	Medium impact	Strong impact
Constant throughout the whole study	◯	◯	◯	◯
Non-constant throughout the study: Period 1	◯	◯	◯	◯
Non-constant throughout the study: Period 2	◯	◯	◯	◯
Non-constant throughout the study: Period 3	◯	◯	◯	◯
Non-constant throughout the study: Period 4	◯	◯	◯	◯
Non-constant throughout the study: Period 5	◯	◯	◯	◯

214. **If the intensity of impact changed during the study period, indicate the start and end of PERIOD 1:**

year start, year end. E.g.: 1990, 1995

____

215. **If the intensity of impact changed during the study period, indicate the start and end of PERIOD 2:**

year start, year end. E.g.: 1990, 1995

____

216. **If the intensity of impact changed during the study period, indicate the start and end of PERIOD 3:**

year start, year end. E.g.: 1990, 1995

____

217. **If the intensity of impact changed during the study period, indicate the start and end of PERIOD 4:**

year start, year end. E.g.: 1990, 1995

____

218. **If the intensity of impact changed during the study period, indicate the start and end of PERIOD 5:**

year start, year end. E.g.: 1990, 1995

____

219. **Comments**

Harbours/marinas/navigation

220. **Estimate the intensity of the impact of harbours/marinas/navigation at the site:**
If intensity was constant or null throughout the whole study, fill in the first row only. If intensity changed during the study period, identify periods of constant impact (maximum 5 periods) and provide an estimate of the impact in each of the identified periods (rows from 2 to 6)
Mark only one oval per row.

	Null	Weak impact	Medium impact	Strong impact
Constant throughout the whole study				
Non-constant throughout the study: Period 1				
Non-constant throughout the study: Period 2				
Non-constant throughout the study: Period 3				
Non-constant throughout the study: Period 4				
Non-constant throughout the study: Period 5				

221. **If the intensity of impact changed during the study period, indicate the start and end of PERIOD 1:**
year start, year end. E.g.: 1990, 1995

222. **If the intensity of impact changed during the study period, indicate the start and end of PERIOD 2:**
year start, year end. E.g.: 1990, 1995

223. **If the intensity of impact changed during the study period, indicate the start and end of PERIOD 3:**
year start, year end. E.g.: 1990, 1995

224. **If the intensity of impact changed during the study period, indicate the start and end of PERIOD 4:**
year start, year end. E.g.: 1990, 1995

225. **If the intensity of impact changed during the study period, indicate the start and end of PERIOD 5:**

year start, year end. E.g.: 1990, 1995

226. **Comments**

Sources of pollution/nutrients/warm water (e.g. from industrial sites)

227. **Estimate the intensity of the impact of pollution/nutrients/warm water at the site:**

If intensity was constant or null throughout the whole study, fill in the first row only. If intensity changed during the study period, identify periods of constant impact (maximum 5 periods) and provide an estimate of the impact in each of the identified periods (rows from 2 to 6)
Mark only one oval per row.

	Null	Weak impact	Medium impact	Strong impact
Constant throughout the whole study	⬭	⬭	⬭	⬭
Non-constant throughout the study: Period 1	⬭	⬭	⬭	⬭
Non-constant throughout the study: Period 2	⬭	⬭	⬭	⬭
Non-constant throughout the study: Period 3	⬭	⬭	⬭	⬭
Non-constant throughout the study: Period 4	⬭	⬭	⬭	⬭
Non-constant throughout the study: Period 5	⬭	⬭	⬭	⬭

228. **If the intensity of impact changed during the study period, indicate the start and end of PERIOD 1:**

year start, year end. E.g.: 1990, 1995

229. **If the intensity of impact changed during the study period, indicate the start and end of PERIOD 2:**

year start, year end. E.g.: 1990, 1995

230. **If the intensity of impact changed during the study period, indicate the start and end of PERIOD 3:**

year start, year end. E.g.: 1990, 1995

231. **If the intensity of impact changed during the study period, indicate the start and end of PERIOD 4:**

year start, year end. E.g.: 1990, 1995

232. **If the intensity of impact changed during the study period, indicate the start and end of PERIOD 5:**

year start, year end. E.g.: 1990, 1995

233. **Comments**

Additional pressures and major changes at the site

234. **Did other major changes occur at the site and its surroundings that could have affected the biodiversity trends during the study period? (e.g. additional pressures, changes in nutrient inputs/deposition, changes in levels of acidification, parasite/desease outbreaks, changed pressure by predators, increased/decreased availability of resources, etc...)** *

Mark only one oval.

- Yes
- No
- Don´t know

235. **If yes, explain WHAT happened and WHEN**

Overall site assessment

236. **Estimate the quality (=naturalness) of the site:**

If quality was constant throughout the whole study, fill in the first row only. If quality changed during the study period, identify periods of constant quality (maximum 5) and provide an estimate of the quality in each of the identified periods (rows from 2 to 6)

Mark only one oval per row.

	Bad	Poor	Moderate	Good	High
Constant throughout the whole study	◯	◯	◯	◯	◯
Non-constant throughout the study: Period 1	◯	◯	◯	◯	◯
Non-constant throughout the study: Period 2	◯	◯	◯	◯	◯
Non-constant throughout the study: Period 3	◯	◯	◯	◯	◯
Non-constant throughout the study: Period 4	◯	◯	◯	◯	◯
Non-constant throughout the study: Period 5	◯	◯	◯	◯	◯

237. **If quality changed during the study period, indicate the start and end of PERIOD 1:**

year start, year end. E.g.: 1990, 1995

____

238. **If quality changed during the study period, indicate the start and end of PERIOD 2:**

year start, year end. E.g.: 1990, 1995

____

239. **If quality changed during the study period, indicate the start and end of PERIOD 3:**

year start, year end. E.g.: 1990, 1995

____

240. **If quality changed during the study period, indicate the start and end of PERIOD 4:**

year start, year end. E.g.: 1990, 1995

____

241. **If quality changed during the study period, indicate the start and end of PERIOD 5:**

year start, year end. E.g.: 1990, 1995

____

242. **Comments**

____

____

____

____

____

Data availability - Water temperature

243. **Are water temperature data available for the whole study period?**

Mark only one oval.

- Yes
- No

Thank you!

Powered by Google Forms
