# Supplementary material for: Meta-analysis of multidecadal biodiversity trends in Europe
Source: Nat Commun. 2020 Jul 13;11:3486. doi: 10.1038/s41467-020-17171-y (PMC7359034; doi:10.1038/s41467-020-17171-y)
Supplement: Supplementary file 3 — Description of Additional Supplementary Files [file 41467_2020_17171_MOESM3_ESM.docx]

Description of Additional Supplementary Files

**Title:** Supplementary Data 1

**Description:** The file reports the information related to each studied time series, including its location, the time span that it covers, and the link to the raw data.
