# Supplementary material for: Meta-analysis of multidecadal biodiversity trends in Europe
Source: Nat Commun. 2020 Jul 13;11:3486. doi: 10.1038/s41467-020-17171-y (PMC7359034; doi:10.1038/s41467-020-17171-y)
Supplement: Supplementary file 5 — Reporting Summary [file 41467_2020_17171_MOESM5_ESM.pdf]

nature research

Corresponding author(s): Francesca Pilotto, Peter Haase

Last updated by author(s): 04.06.2020

Reporting Summary

Nature Research wishes to improve the reproducibility of the work that we publish. This form provides structure for consistency and transparency in reporting. For further information on Nature Research policies, see Authors & Referees and the Editorial Policy Checklist.

Statistics

For all statistical analyses, confirm that the following items are present in the figure legend, table legend, main text, or Methods section.

n/a	Confirmed	
☐	☒	The exact sample size (*n*) for each experimental group/condition, given as a discrete number and unit of measurement
☒	☐	A statement on whether measurements were taken from distinct samples or whether the same sample was measured repeatedly
☐	☒	The statistical test(s) used AND whether they are one- or two-sided<br>*Only common tests should be described solely by name; describe more complex techniques in the Methods section.*
☐	☒	A description of all covariates tested
☐	☒	A description of any assumptions or corrections, such as tests of normality and adjustment for multiple comparisons
☐	☒	A full description of the statistical parameters including central tendency (e.g. means) or other basic estimates (e.g. regression coefficient) AND variation (e.g. standard deviation) or associated estimates of uncertainty (e.g. confidence intervals)
☐	☒	For null hypothesis testing, the test statistic (e.g. *F*, *t*, *r*) with confidence intervals, effect sizes, degrees of freedom and *P* value noted<br>*Give P values as exact values whenever suitable.*
☒	☐	For Bayesian analysis, information on the choice of priors and Markov chain Monte Carlo settings
☒	☐	For hierarchical and complex designs, identification of the appropriate level for tests and full reporting of outcomes
☐	☒	Estimates of effect sizes (e.g. Cohen's *d*, Pearson's *r*), indicating how they were calculated

Our web collection on statistics for biologists contains articles on many of the points above.

Software and code

Policy information about availability of computer code

Data collection	no software used
Data analysis	We used the R packages: codyn (version 2.0.0), metafor (2.1-0), glmulti (version 1.0.7), vegan (version 2.5-4), reshape2 (version 1.4.3), R.utils (version 2.8.0), sp (version 1.3-1), raster (version 2.8-19), ncdf4 (version 1.17), rgdal (version 1.4-3), rgeos (version 0.4-2), RColorBrewer (version 1.1-2), ggplot2 (version 3.1.1), ggpubr (version 0.2.5). The link to the used R-codes (https://github.com/Biodiversity-trends-in-Europe-ILTER/R-code) is indicated in the manuscript under 'code availability'.

For manuscripts utilizing custom algorithms or software that are central to the research but not yet described in published literature, software must be made available to editors/reviewers. We strongly encourage code deposition in a community repository (e.g. GitHub). See the Nature Research guidelines for submitting code & software for further information.

Data

Policy information about availability of data

All manuscripts must include a data availability statement. This statement should provide the following information, where applicable:

- Accession codes, unique identifiers, or web links for publicly available datasets
- A list of figures that have associated raw data
- A description of any restrictions on data availability

The data underlying all analyses are made publicly available on the DEIMS-SDR registry (Dynamic Ecological Information Management System - Site and dataset registry; https://deims.org/) which is the central note of the European Long-term Ecological Research (eLTER) network and in b2share. The links to all 161 datasets are indicated in Supplementary data. Some of the 161 datasets are immediatly accessible, while the others will be accessible after an embargo of six months, on 1st January 2021.
Data underlying all figures are provided as Source data.
The R code used to analyse the data and to produce the figures is available at the dedicated GitHub repository (https://github.com/Biodiversity-trends-in-Europe-ILTER/R-code), as indicated in the manuscript under 'code availability'.

Field-specific reporting

Please select the one below that is the best fit for your research. If you are not sure, read the appropriate sections before making your selection.

☐ Life sciences ☐ Behavioural & social sciences ☒ Ecological, evolutionary & environmental sciences

For a reference copy of the document with all sections, see nature.com/documents/nr-reporting-summary-flat.pdf

Ecological, evolutionary & environmental sciences study design

All studies must disclose on these points even when the disclosure is negative.

Study description	The study is based on existing data. We compiled 161 long-term (minimum 15 years) biomonitoring time series from 115 sites, mostly members of the International Long-Term Ecological Research network, in 21 European countries, for nine bioregions, three realms and eight taxonomic groups. First, we analyzed each time series separately to quantify time-series-specific biodiversity trends. Second, we combined the effect sizes of the different time-series-specific biodiversity trends, using a meta-analysis approach to synthesize the overall trends and identify common patterns and drivers.
Research sample	The studied dataset includes: 161 long-term (spanning between 15 and 91 years, median: 20 years) biomonitoring time series from 115 sites, in 21 European countries, for nine bioregions, three realms and eight taxonomic groups. Data creators and/or data owners for all studied 161 time series are indicated at the respective link to the data repository, as indicated in Supplementary data.
Sampling strategy	The study is based on existing data. We circulated a call for biodiversity data within the ILTER (International Long-Term Ecological Research network) network and additional partners to fill in geographical gaps. The criteria for data selection were: (1) each time series covers at least 15 years, (2) with at least ten survey events during that time, (3) sampling occurred at the same site (no space-for-time substitution), and (4) survey method, seasonal and taxonomic resolution were consistent throughout the whole study period for each time series.
Data collection	The study is based on existing biomonitoring data. Survey methods and season varied among time series, but were kept constant within each time series throughout the entire study period. Taxonomic resolution was constant for each time series throughout the entire study period. Data creators and/or data owners for all studied time series are indicated at the respective link to the data repository, as indicated in Supplementary data.
Timing and spatial scale	The time span of the studied time series ranged between 15 and 91 years (median: 20), with starting years ranging between 1920 and 2003 (median: 1994) and end years between 2005 and 2018 (median: 2015). As the study is based on existing biomonitoring data, we did not decided specific start or end dates, rather we used all available time series that met the criteria listed in the section "sampling strategy".<br>Spatial scale: Europe.
Data exclusions	Time series were excluded if they did not satisfy the data selection criteria reported above (see Sampling strategy). No further exclusion criteria were applied.
Reproducibility	The study is based on existing observational data (biodiversity monitoring), no experiments were carried out.
Randomization	Randomization was used for a sensitivity analysis, to assess the effect of the unbalanced design on the results of the meta-analysis mixed models. We randomly sampled the over-represented biotic groups within each biogeoregion to the maximum number of time series available for the other biotic groups within that biogeoregion. We repeated the random sampling 5 times and, with those data, we re-run the meta-analysis mixed models for biogeoregions, as described in the main text. The same procedure was applied for the over-represented biogeoregions within each biotic groups. The procedure is fully described in Supplementary notes.
Blinding	Blinding is not relevant for this study because it is an observational study (biodiversity monitoring), no experiments were carried out.

Did the study involve field work? ☐ Yes ☒ No

Reporting for specific materials, systems and methods

We require information from authors about some types of materials, experimental systems and methods used in many studies. Here, indicate whether each material, system or method listed is relevant to your study. If you are not sure if a list item applies to your research, read the appropriate section before selecting a response.

Materials & experimental systems

n/a	Involved in the study
☒	☐ Antibodies
☒	☐ Eukaryotic cell lines
☒	☐ Palaeontology
☒	☐ Animals and other organisms
☒	☐ Human research participants
☒	☐ Clinical data

Methods

n/a	Involved in the study
☒	☐ ChIP-seq
☒	☐ Flow cytometry
☒	☐ MRI-based neuroimaging
